# Performance of Self-Compacting Mortars Using Ground Seashells as Recycled Sand

**DOI:** 10.3390/ma18020418

**Published:** 2025-01-17

**Authors:** Ágata González-Caro, Antonio Manuel Merino-Lechuga, David Suescum-Morales, Enrique Fernández-Ledesma, José María Fernández-Rodríguez, José Ramón Jiménez

**Affiliations:** 1 Department of Inorganic Chemistry, University of Córdoba, E.P.S of Belmez, Avenida de la Universidad s/n, E-14240 Córdoba, Spain; q32gocaa@uco.es; 2 Department of Construction Engineering, University of Córdoba, E.P.S of Belmez, Avenida de la Universidad s/n, E-14240 Córdoba, Spain; ammlechuga@uco.es (A.M.M.-L.); p02sumod@uco.es (D.S.-M.); efledesma@uco.es (E.F.-L.)

**Keywords:** industrial by-product, milled seashells, self-compacting mortar, siliceous filler, circular economy

## Abstract

The findings highlight the potential for broadening the use of shell aggregates in construction applications. This research investigated the viability of incorporating milled *Acanthocardia tuberculata* seashells as fine sand replacements for natural calcareous sand in the production of self-compacting mortar. These results highlight a promising avenue for coastal industries to reduce waste while enhancing the durability of construction materials. Mortar mixtures containing recycled seashell aggregates exhibit superior overall performance compared with those using natural sand in terms of durability, although there is a slight reduction in workability and mechanical strength. Three replacement levels of natural limestone sand (0%, 50%, and 100%) with seashell-based fine aggregates were studied, along with three different powdered/sand ratios. The fresh properties of the mixtures were assessed for workability, whereas the hardened specimens were analyzed using an X-ray technique, thermogravimetry, and differential thermal analysis. Key performance and durability properties, including compressive and flexural strengths, bulk density, porosity, water absorption, dimensional stability, and mercury intrusion porosimetry at 28 days of hardening, were also evaluated. Overall, the incorporation of *Acanthocardia tuberculata* seashells into cementitious materials supports the principles of the circular economy, providing both environmental and performance advantages.

## 1. Introduction

The growing demand for quarried aggregates in mortar and concrete production has resulted in environmental issues, including alterations to riverbeds, depletion of sand reserves, and harm to ecosystems in various regions [1]. The extraction of traditional aggregates, typically obtained through energy-intensive mining processes, contributes significantly to environmental degradation, including pollution and depletion of natural reserves. The worldwide demand for aggregates in the construction industry was estimated to range from 25.9 to 29.6 billion tons in 2012 [2]. According to a recent report by the U.S.-based independent research firm Persistence Market Research, global consumption of construction aggregates is expected to rise significantly, reaching 62.9 billion tonnes by the end of 2024, compared to 43.3 billion tonnes in 2016 [3].

To address these problems, the European Union has advocated a circular economic approach that promotes the use of waste as alternative materials in various industries [4].

Simultaneously, the shellfish aquaculture industry has seen substantial growth, from producing 1 million tons in 1950 to around 87.5 million tons in 2020. According to a report by the FAO (Food and Agriculture Organization), in 2022, for the first time in history, aquaculture overtook capture fisheries as the main producer of aquatic animals. Global aquaculture production reached a record 130.9 million tons [5]. By 2030, the amount of waste generated from aquaculture and fisheries is expected to exceed 106 million tons [6]. Despite the large volume of seashells generated, they are often discarded to landfills because of their low commercial value, which poses significant environmental and health risks [1,6,7]. For instance, the decomposition of organic matter in intensive oyster farming leads to the production of hydrogen sulfide, a hazardous product of sulfate reduction that accumulates in landfills [8]. As the amount of these waste materials increases, alternatives are being explored to repurpose seashells that are primarily composed of CaCO_3_ in the form of vaterite and aragonite [9,10]. Their applications include filtration media [11], construction materials [12,13,14,15], and even as catalysts [16].

Employing seashell-derived materials as an alternative to quarried aggregates in mortar and concrete has been a key area of research in the construction sector. Researchers have examined the performance, durability, and properties of these modified materials [7,17,18]. Tayeh et al. [19] found that seashell fillers can serve as partial replacements for cement with a CaCO_3_ content exceeding 90%, making them suitable for producing limestone Portland cement. Similarly, Kuo et al. [14] observed that replacing up to 20% of sand with crushed oyster shells had a minimal impact on the strength but reduced the workability of the mix. Other studies, such as those by González-Caro et al. [20], Liao et al. [21], and Liao et al. [22], have explored the use of various seashell types, reporting strengths comparable to conventional mixtures, but with some reductions in workability and increases in water absorption (WA) owing to the angular geometry and porosity of the shells.

A similar conclusion was reached by Zhang et al. [18], who explored the mechanical performance of cellular concrete with the addition of sea snail seashells. They reported a decline in compressive strength as the proportion of seashell waste increased, dropping from 35.8 MPa to 9.4 MPa. This reduction was attributed to the inherent weakness of seashells compared to crushed stone despite their solid structure.

Martínez-García et al. [10] examined the potential of mussel shells as substitutes for natural limestone sand and gravel in concrete. The study revealed that the increased WA and irregular geometry of the shells caused air entrapment, thereby reducing compressive strength.

Yang et al. [23] on the other hand, observed that replacing up to 20% of fine aggregates with oyster shells resulted in higher early-age compressive strength, but a decrease at later ages (56 days), attributed to stress accumulation on the weaker shells.

The primary challenge in using seashells as aggregates is their high porosity and uneven particle geometry, which can result in poor performance properties and consistency owing to increased WA. However, the incorporation of shells does not negatively affect cement hydration and offers a potential solution to the problem of shell waste accumulation while supporting circular economy practices.

Self-compacting concrete (SCC), an innovative compound that simplifies placement and improves surface quality, strength, and durability, has been widely adopted in construction. By modifying the self-compacting mortar (SC mortar) phase, Billberg [24] and Nepomuceno et al. [25] sought to optimize its performance. However, SCC is more expensive than conventional concrete, leading to the use of additional materials such as crushed seashells to reduce costs and enhance durability.

Considering the environmental benefits and potential material savings, this study investigated the viability of replacing natural calcareous aggregate (NA) with recycled sand from *Acanthocardia tuberculata* seashells (ATS) for SC mortar manufacturing. ATS is classified as a cockle species characterized by 20–24 pronounced ridges and refined circular lines. To date, no study has fully explored the use of ATS in SC mortar, making this a significant contribution to the field. The results of this comprehensive analysis of the durability and physicochemical and mechanical properties of ATS-based SC mortar will provide new insights into its potential as an SC mortar, aligning with the European Union’s push for a circular economy.

The objective of this study is to evaluate the partial (50%) and complete (100%) replacement of natural aggregates with shell aggregates in SC mortars incorporating siliceous fillers. The study also investigates the influence of the siliceous filler on the setting behavior of these mortars. X-ray diffraction and thermogravimetric analysis techniques have been used. Additionally, the mechanical performance and durability properties of the mortars were assessed. The findings highlight the potential for broadening the use of shell aggregates in construction applications.

The use of shells of the species *Acanthocardia tuberculata* is a great alternative to the natural materials normally used in the construction sector. This study offers a sustainable alternative by preventing the accumulation of ATS in landfills and reintegrating it into the construction sector, thereby contributing to ongoing innovation and sustainability in the industry.

## 2. Materials and Methods

### 2.1. Materials

In this study, the SC mortar specimens used as references were produced using a mixture of calcareous natural aggregates: NA-0/3 and NA-0/6. NA-0/3 contains 5.2% particles lower than 0.063 mm. NA-0/6 contains 23.3% particles lower than 0.063 mm. The 50/50 mixture of both contains 12.2% particles lower than 0.063 mm. A commercial siliceous filler (SF) from Lorda and Roig S.L. (Barcelona, Spain) was used as a mineral additive. All the experimental procedures used ordinary Portland cement (CEM II A/L 42.5 R) from Votorantim Cementos, Pontevedra, Spain. To create a self-compacting mixture, a superplasticiser (Sp) was used.

To determine the effect of recycled sand from ATS seashells on the reference SC mortar properties, two volumetric substitution ratios were used: 50% and 100%. ATS seashells were acquired from a canning organization in Málaga, Spain. To ensure that seashells did not contain any salts or organic material, they were cleaned and air-dried at 135 °C for 32 min. The authors of this paper demonstrated the need for this cleaning in previously published articles [20]. To ensure that the ATS seashell sand had a particle dimension similar to both calcareous natural aggregates, a ball-milling study was performed. ATS aggregate contains 12.6% particles lower than 0.063 mm. In this study, the number of balls and laps of the mill were modified until a particle size distribution curve similar to that of natural aggregates was obtained. This study is explained in more detail in previous research carried out according to the writers [26]. The selected milling method consisted of 2000 laps and 11 balls.

The WA of the ATS sand was 2.19%, and its density was 2.72 g/cm^3^. The average WA of the two types of NA (NA-0/3 and NA-0/6) was 2.09%, and the average density was 2.63 g/cm^3^.

### 2.2. SC Mortar Design

An SC mortar formulation was developed using the Nepomuceno method [25]. In this approach, CEM and SF were categorized as ‘powdered materials’, while NA-0/3, NA-0/6, and ATS sand were classified as ‘fine sand’. The method used is explained in more detail in previously published articles [20,26].

Table 1 provides details on the nomenclature, mixing ratios (kg/m^3^), and workability characteristics of each SC mortar tested. The workability properties were assessed using the absolute volume ratios of powdered materials and fine sand, with a fixed w/c ratio of 0.49.

Control mortars were produced by combining 50% NA-0/3 and 50% NA-0/6. Three test mixtures, labeled Cont-1, Cont-2, and Cont-3, were evaluated. The control specimens were manufactured with Vp/Vs ratios of 0.6, 0.7, and 0.8, for Cont-1, Cont-2, and Cont-3, respectively. Substitution ratios of 50% and 100% ATS sands were evaluated across the three control mixtures.

Within all groups, the CEM/SF ratio of 1.67 was kept. For Groups-1 and -2, the Sp/p % ratios were 0.84. However, for Group-3, the Sp/p % ratio had to be lowered to a value of 0.69 to maintain the limits of workability, between 220 and 260 mm for the spread test and between 7 and 11 s for the flow velocity test.

The values that satisfied the workability criteria were those that satisfied the workability requirements and were experimentally assessed using slump flow and V-funnel tests. The slump flow test measured the relative spread area (Gm), whereas the V-funnel test determined the relative flow velocity (Rm). Both tests are described in the Nepomuceno method and in the EFNARC guidelines [25,27]. The target values for a mixture to be considered self-compacting are between 4.84 and 6.76 for Gm and between 7 and 11 s for Rm.

The kneading process was carried out as follows: The solid components were primarily mixed at a slow pace for 30 s using a conventional mortar mixer. Water and the superplasticiser were then incorporated, and the blend was homogenized for over 6 min at a slow pace. The blender was then paused for 2 min to ensure proper blending of all ingredients. The mixture was stirred again at a slow pace for an additional 2 min. Prismatic molds measuring 4 × 4 × 16 cm were prepared for each type of SC mortar avoiding any compression or vibratory energy. The samples were taken out from the moulds 24 h later and placed in a curing humidity chamber.

### 2.3. Test Methods

X-ray emission analysis was conducted using a Rigaku instrument (Tokyo, Japan). The identification of mineral components in the principal materials and specimen was performed using diffraction analysis via X-ray (XRD) with a D8 Discover A25 instrument with CuK = 1.54050 Å; 40 kV; 30 mA, Bruker (Billerica, MA, USA). Additionally, gravimetric thermal (TGA) and differential thermal analysis (DTA) were realized with a Setaram Setsys Evolution 16/18 device (Setaram, Caluire-et-Cuire, France).

The performance properties of the specimens were determined through compressive and flexural strength tests. Flexural strength measurements were conducted on three prismatic samples of each mixture, sized 4 × 4 × 16 cm.

Dry bulk density (DBD), WA, and accessible porosity to water (APW) were assessed in compliance with UNE-EN 83980 [28]. Mercury porosimetry analysis (MIP) was employed to determine the pore volume of the specimens. The Poremaster 60GT device, Quantachrome Instrument (Boynton Beach, FL, USA) was used for MIP [29]. Shrinkage measurements were carried out with an IBERTEST Universal Shrinkage Meter according to UNE 83831:2021 [30]. Capillary water absorption tests were executed following UNE-EN 1015-18:2003 [31].

## 3. Results and Discussion

### 3.1. Chemical Assessment of Primary Materials

The materials used were previously characterized by the authors [26], with the exception of SF. The density of the SF was 2600 kg/m^3^. The elements obtained by XRF and expressed in the form of oxides indicated that CaO was the predominant element in all primary materials, except for SF. The main elements found in CEM were 70.03% CaO and 15.58% SiO_2_ [32,33]. The CaO levels in the NA (98.31% for NA-0/3 and 60.44% for NA-0/6) aligned with their limestone composition, supporting the suitability of replacing NAs with ATS sands. NA-0/6 also exhibited a higher MgO content than NA-0/3. The CaO percentage of the ATS sand was 96.81%, which varied depending on the shell type and thermal treatment [20,34]. The minor components of the ATS sands included SiO_2_ (1.25%), Al_2_O_3_ (0.15%), and Fe_2_O_3_ (0.11%) [7,14,18].

**Figure 1:**
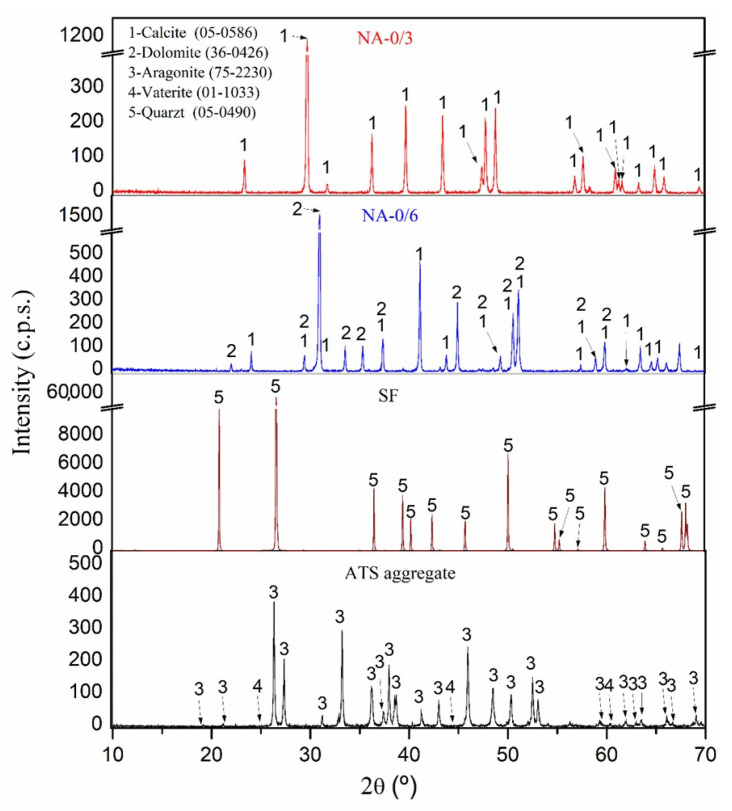
XRD patterns of primary materials.

Figure 1 illustrates the mineral structure of the primary materials, as determined by XRD analysis. The primary phase in both NA-0/3 and NA-0/6 was calcium carbonate in the form of calcite [35]. NA-0/6 also exhibited a dolomite (CaMg(CO_3_)_2_) [35] phase, which was confirmed by XRF as one of its major components. Analogous findings were observed in previous studies focusing on analogous aggregates [20]. The principal phases detected in the ATS sands were aragonite [35] and vaterite [35], which are both polymorphs of calcium carbonate (CaCO_3_). The quartz (SiO_2_) [35] phase was found in the SF.

**Figure 2:**
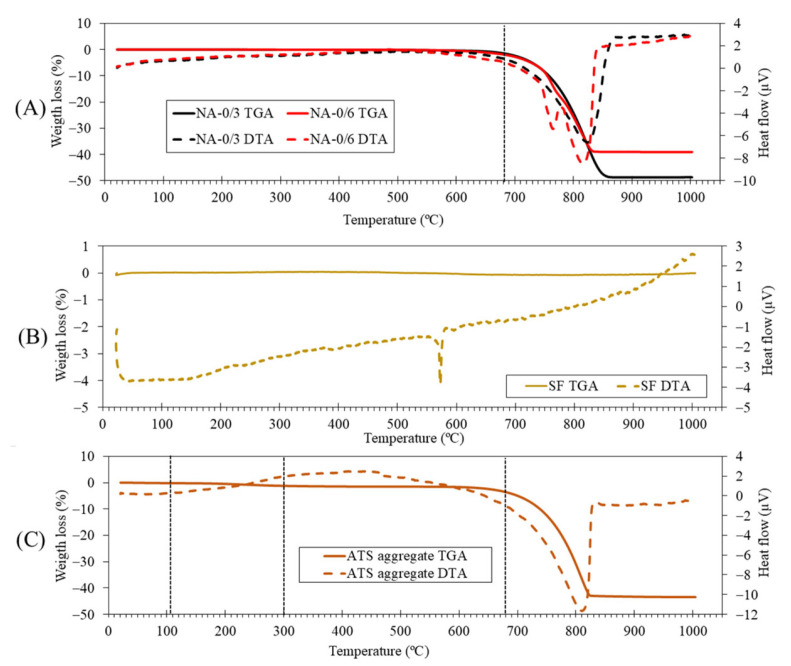
TGA–DTA of primary materials: (**A**) NA-0/6 and NA-0/3, (**B**) SF, and (**C**) ATS sand.

Figure 2 shows the TGA and DTA of the primary materials. For NA-0/3 (A), an endothermic peak appeared around 830 °C, linked to the degradation of calcium carbonate. This was consistently associated with the calcite phase identified in the XRD analysis (Figure 1) and the calcium oxide detected in the XRF results. Regarding NA-0/6 (A), along with an endothermic peak at 830 °C for calcite decomposition, another peak was detected at 760 °C, indicating the degradation of dolomite, as detected by XRD (Figure 1). The existence of Mg, confirmed by XRF, further supported this hypothesis. These results corroborated earlier research conclusions [36,37,38]. The TGA of SF (B) revealed its purity because no weight loss was detected. The SF DTA curve showed a peak at 570 °C that identified the crystalline phase change in quartz, which is denoted as the conversion of the α-phase to the β-phase [39].

In the case of the ATS sand (C), different stages were recognized: (i) loss of humidity occurred up to 105 °C, (ii) highly evaporative organic matter was eliminated from 105 °C to 300 °C, and (iii) hardier organic matter was lost between 400 °C and 500 °C. Additionally, the DTA curve revealed multiple thermal effects connected to the conversion of vaterite and aragonite into calcite, where the effects were endothermic for aragonite and exothermic for vaterite [40]. An endothermic peak at 820 °C was observed between 600 °C and 850 °C, corresponding to the decomposition of calcium carbonate, consistent with studies by Barros et al. [41] and Mohamed et al. [42] for mussel and cockle shells, respectively.

### 3.2. SEM of Aggregates

**Figure 3:**
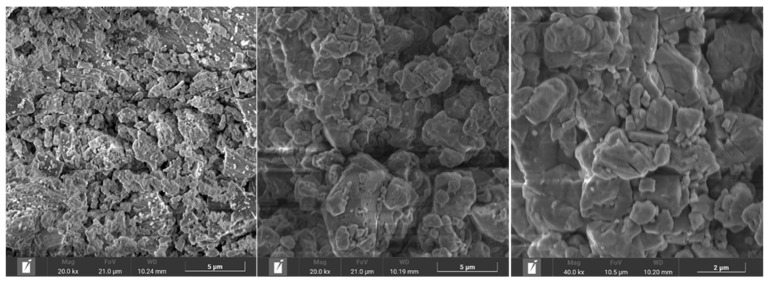
*Acanthocardia tuberculata* aggregate (**left**), natural aggregate, NA-0/3 (**center**) and natural aggregate, NA-0/6 (**right**) images by SEM.

Figure 3 shows SEM images of the ATS sands (left), NA-0/3 (center), and NA-0/6 (right). The ATS sand was more porous and had a more uneven and angular surface than NA-0/3 and NA-0/6, which provides a good bond between the cement paste and the ATS sands. The surfaces of both the natural aggregates were smooth with rounded edges. This is consistent with the higher WA of ATS sands compared with the average absorption of natural aggregates (Section 2.1). Hadjadj et al. [43] carried out an SEM analysis of ground oyster shells and found that the shells had a rough surface and angular geometry.

### 3.3. Workability Characteristics of Mixtures

Table 2 provides the workability features of the SC mortar specimens evaluated in this research, utilizing the workability parameters Gm and Rm, as outlined in Section 2.2. A methodical and iterative approach, based on Nepomuceno’s methodology [25] and adhering to EFNARC guidelines [27], was implemented. The acceptable ranges for the mixtures tested were 4.86–5.76 for Gm and 1.00–1.43 s^−1^ for Rm, ensuring compliance with the workability criteria. The results demonstrated excellent performance, as the fresh SC mortar mixtures exhibited neither segregation nor bleeding.

A small decline in both consistency (Gm) and fluidity (Rm) was observed as the proportion of ATS sand increased across all mixture groups. This reduction is linked to the pointed and asymmetrical geometry of ATS particles, as highlighted by SEM analysis (Figure 3). The distinctive geometry causes ATS sand to exhibit behavior akin to coarse aggregates in SCC, resulting in a perception of dryer-like consistency. Similar observations were made by Safi et al. [44] who reported that natural sands, with their more spherical shape, generally improve the fluidity of mortar. Oh et al. [45] examined the workability of mortars containing various shell types as aggregates at substitution levels of 20%, 30%, and 40%. Their results showed a reduction in flowability with increasing substitution ratios, mainly attributed to the shells’ higher WA capacity compared to natural sand.

Hadjadj et al. [43] explored the combined use of seashell powder as a cement additive and granite waste as a substitute for fine aggregates in concrete. Their results showed that incorporating seashell powder reduced both slump flow and paste viscosity, which they attributed to the particle geometry and increased WA of the materials.

To mitigate the issue of dry consistency, superplasticisers were utilized as a corrective measure. In SC mortar, the dosage of superplasticiser was tailored to meet the specific requirements of each mixture, ensuring the desired slump and spread were achieved, as outlined in Section 2.2.

### 3.4. Hardened Properties of the Mixtures

#### 3.4.1. Analysis of Cured Mortar Properties

**Figure 4:**
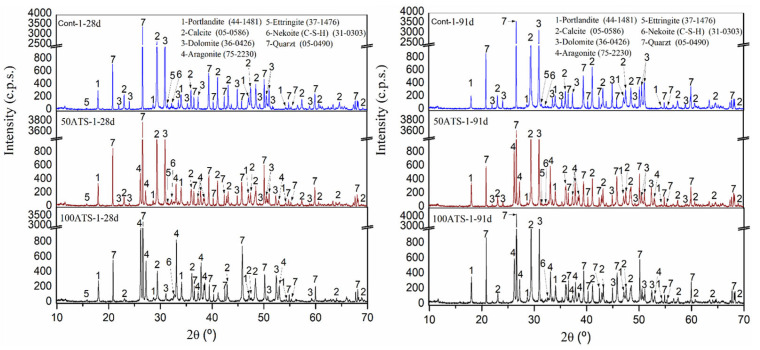
XRD diffractograms of Group-1 of hardened SC mortar at 28 (**left**) and 91 days (**right**) of hardening.

Figure 4 presents the XRD diffractograms of the Group-1 SC mortar specimens at 28 days (left) and 91 days (right) of hardening of Group-1 specimens. The phases observed in the Group-2 and -3 specimens were identical, making it not required to include these graphs. The quartz phase was detected in all the control mortars and in the 50ATS and 100ATS sands. This phase originated from the SF. The primary phase was quartz. Calcite and dolomite appeared in all the control samples. These phases were derived from NA-0/3 and NA-0/6 and were identified using XRD (Figure 1), which revealed that the principal compounds were quartz, calcite, and dolomite. Nekoite, portlandite, ettringite, and calcium silicate hydrate (C-S-H) phases were found. These last phases were formed because of reactions occurring within the cement [20,36,46]. Furthermore, for the 50ATS and 100ATS specimens, the aragonite phase was also observed, which was consistent with the phase observed in the XRD diffractograms of the primary material (Figure 1). A similar phase has been observed by other scientists analyzing seashells [20,34,47].

**Figure 5:**
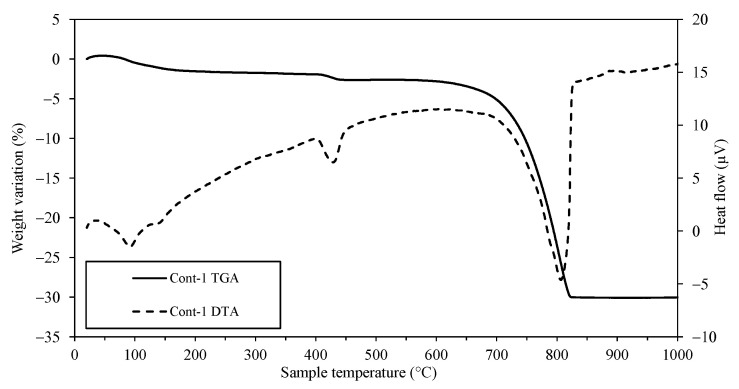
TGA–DTA of Cont-1 at 28 days of hardening.

**Figure 6:**
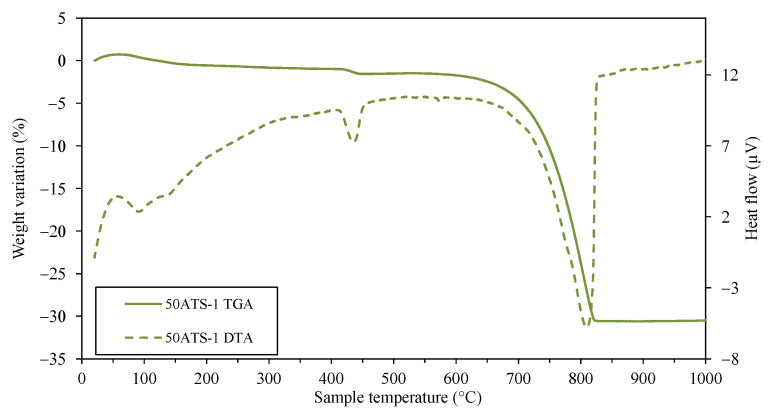
TGA–DTA of 50ATS-1 at 28 days of hardening.

**Figure 7:**
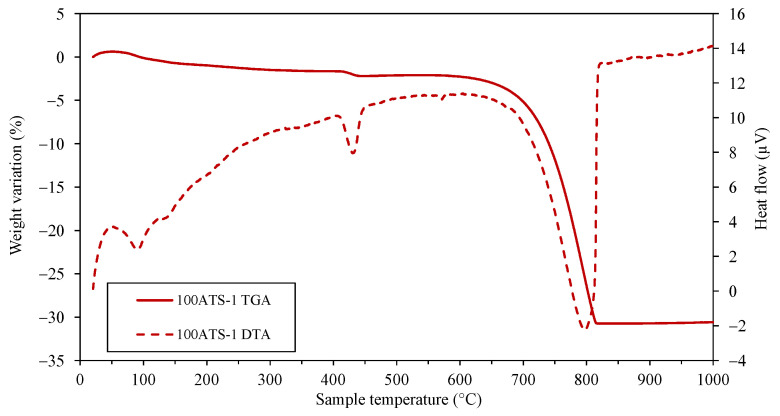
TGA–DTA of 100ATS-1 at 28 days of hardening.

The amount of each phase was roughly indicated by the XRD technique, correlating with the intensity on the *y*-axis. The intensity of calcite increased very slightly for Cont and 50ATS; however, an appreciable change was observed in 100ATS, indicating the possible transformation of the largest amount of portlandite into calcite. The presence of ATS did not reduce typical cement hydration reactions because the intensity of the portlandite phase was not reduced by the addition of ATS sands. These phases were in accordance with the results of the TGA–DTA technique at 28 days of hardening (Figure 5, Figure 6 and Figure 7), where the mineral phases were associated with weight loss across the defined temperature ranges.

Figure 5, Figure 6 and Figure 7 show the TGA–DTA results for Group-1 of the SC mortars analyzed (Cont-1, 50ATS-1, and 100ATS-1, respectively). The phases identified for Groups-2 and -3 were the same as for Group-1. Hence, they did not need to be captured in a additional figure. TGA was conducted to determine the quantity of hydration compounds in the SC mortar. All the mixtures (Cont, 50ATS, and 100ATS) exhibited five distinct stages. The beginning stage, occurring from 25 °C to 105 °C, was associated with water evaporation and partial dehydration of ettringite [45]. The second stage, up to 380 °C, involved the degradation of C-S-H, aluminates, and ettringite [44]. The third stage, around 380 °C, resulted from the dehydroxylation of portlandite [20,46], as seen in the XRD results (Figure 4). Stage four, occurring between 500 °C and 850 °C, resulted from the breakdown of calcium carbonate [45], generated during cement setting and derived from the main materials used. The fifth and final stage, from 850 °C to 1000 °C, is related to the elimination of the remaining -OH groups.

The TGA–DTA and XRD analyses showed that the ATS sand seashells did not affect the setting process of the cement paste.

#### 3.4.2. Mechanical Strength

**Figure 8:**
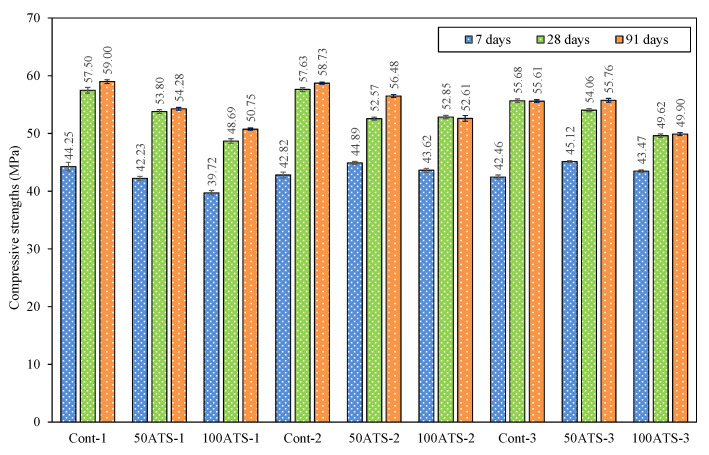
Compressive strengths of specimens studied at 7, 28, and 91 days of hardening.

**Figure 9:**
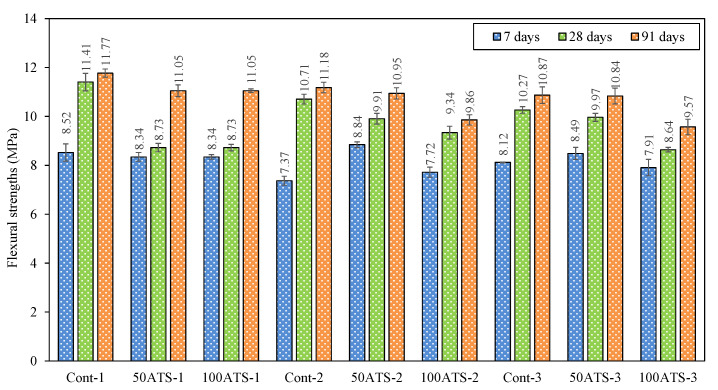
Flexural strengths of specimens studied at 7, 28, and 91 days of hardening.

The mechanical behavior of the three SC mortar groups is illustrated in Figure 8 and Figure 9 after 7, 28, and 91 days of hardening.

The control mixtures obtained compressive strength values of 57.50 ± 0.50 MPa, 57.63 ± 0.32 MPa, and 55.68 ± 0.33 MPa, respectively. The compressive strength decreased slightly when Vp/Vs = 0.8. The dosage of Group-3 had a higher amount of sand aggregate; therefore, the strength might be slightly affected by the higher number of voids formed inside it. A decrease in compressive strength was noted with a rise in ATS sand content. The decreases in Group-1 were 6.43% (Cont-1 vs. 50ATS-1) and 15.32% (Cont-1 vs. 100ATS-1). In Group-2, the decreases were 8.78% (Cont-2 vs. 50ATS-2), 8.29% (Cont-2 vs. 100ATS-2), and in Group-3, they were 2.91% (Cont-3 vs. 50ATS-3), and 10.88% (Cont-3 vs. 100ATS-3). Consequently, the compressive strength decreased slightly as the proportion of ATS sand replacement increased. The porous surface and geometry of the particles (Figure 6) can affect the filling of SC mortar. When Vp/Vs = 0.7, there was a balance between the powder and sand materials. An 8.29% decrease in strength was observed for 100ATS relative to the control mortars. These results improved the results achieved by Suarez et al. [48]. They observed reductions in compressive and flexural strengths with increased ATS content. For mixtures containing 5% and 15% ATS, compressive strength reductions were less than 1% and 6%, respectively. However, for samples with 10% ATS, the strength decreased by approximately 12% compared to the reference mortar using natural sand. This was associated with the high WA of the shells, which lowered the w/c ratio and impaired complete cement hydration. Notably, this study is among the few focusing on this specific kind of shell. On the other hand, Oh et al. [45] examined the effects of different kinds of shells as aggregates in cement-based materials. This author used cockle, a species similar to the one in this research, substituting natural sand with 20%, 30%, and 40% seashell sand. They observed a consistent decrease in compressive strength with greater replacement levels linked to the surface characteristics of the shells.

In a previous investigation conducted by the authors using commercial limestone filler with ATS sand [26] and filler from ATS seashells [20] in SC mortar instead of an SF, the compressive strengths decreased with the use of ATS seashells. These decreases were 29.43%, 16.84%, and 2.29% for Vp/Vs ratios of 0.6, 0.7, and 0.8, respectively. This phenomenon is primarily linked to the previously discussed factors. Thus, the decrease in the compressive strength was primarily attributed to the higher WA and uneven geometry of the seashell particles. This aligned with the findings concerning the fresh properties of the specimens (Table 2), where increases in ATS sands led to reduced consistency and fluidity. It is essential to emphasize that, based on our understanding, no earlier research has investigated the use of ATS as sand in SC mortars.

The flexural strength (Figure 9) also decreased with the incorporation of the ATS sand. In Group-1, there were decreases of 23.48% (Cont-1 vs. 50ATS-1) and 23.48% (Cont-1 vs. 100ATS-1); in Group-2, there were decreases of 7.47% (Cont-2 vs. 50ATS-2) and 12.79% (Cont-2 vs. 100ATS-2); and in Group-3, there were decreases of 2.92% (Cont-3 vs. 50ATS-3) and 15.87% (Cont-3 vs. 100ATS-3).

#### 3.4.3. Dry Bulk Density, Accessible Porosity of Water, and Water Absorption

**Figure 10:**
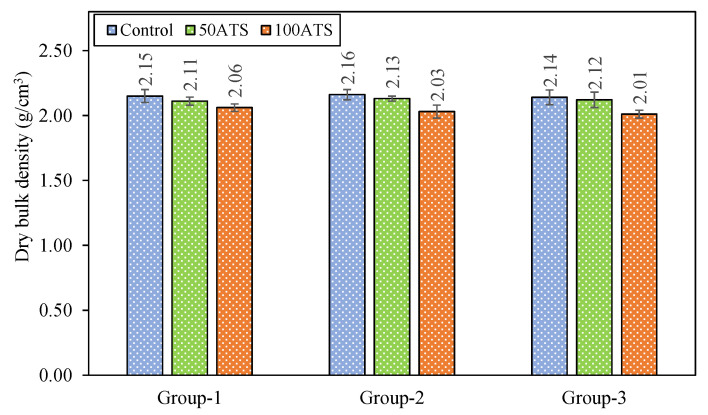
DBD (g/cm^3^) of specimens studied at 28 days of hardening.

**Figure 11:**
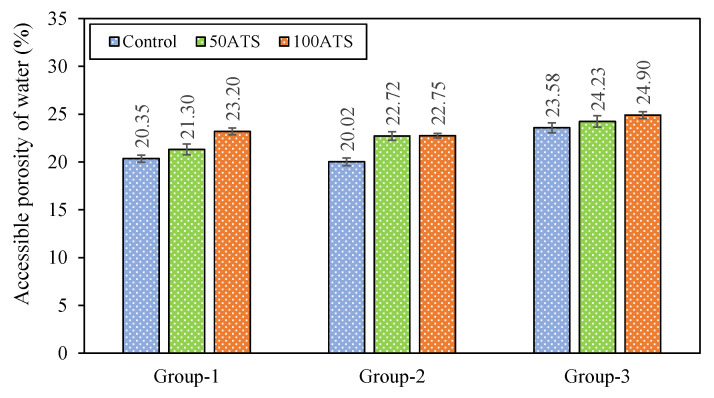
Accessible porosities of water (%) of specimens studied at 28 days of hardening.

**Figure 12:**
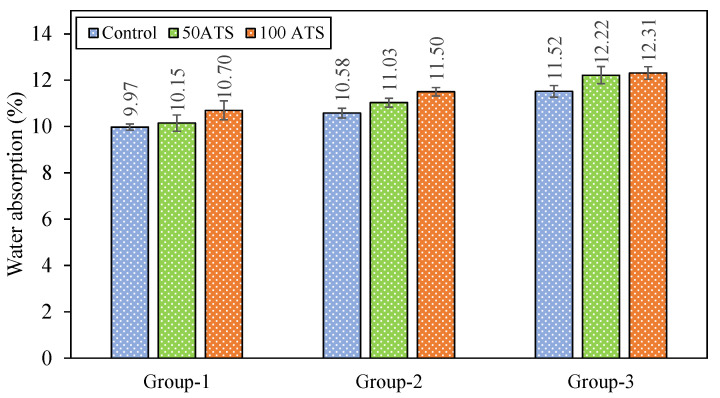
Water absorptions (%) of specimens studied at 28 days of hardening.

DBD (Figure 10), APW (Figure 11), and WA (Figure 12) of the set mortar were assessed after 28 days of setting.

DBD (g/cm^3^) of the specimens in Group-1 decreased by replacing 50ATS and 100ATS sands with 1.86% (Cont-1 vs. 50ATS-1) and 4.19% (Cont-1 vs. 100ATS-1), respectively. In Group-2, the densities decreased by 1.39% (Cont-2 vs. 50ATS-2) and 6.02% (Cont-2 vs. 100ATS-2). In Group-3, the densities decreased by 0.93% (Cont-3 vs. 50ATS-3) and 6.07% (Cont-3 vs. 100ATS-3). The densities of the mixtures made with 100ATS decreased the most compared with those of the control mixtures. Again, for the mixture with Vp/Vs = 0.8, the density was the lowest. This may be because increasing the aggregate versus filler content (Table 1) resulted in a lower densification of the samples. These findings were in line with the results obtained for the compressive strength (Figure 8), where the density decreased, as did the compression.

The uneven geometry of the seashells led to the creation of voids, resulting in decreased density [10,20]. Martínez-García et al. [10] investigated the replacement of natural sand with mussel shell sand in concrete at substitution levels of 25%, 50%, 75%, and 100%. When the substitution rate of mussel shell aggregates rose from 25% to 100%, the density decreased by 5% and 10%, respectively. This effect can be attributed to the flattened and stretched geometry of the particles, which allows air to become trapped. For example, Edalat-Behbahani et al. [49] studied the complete replacement (100%) of natural sand with crushed cockle seashells in concrete and mortars at a w/c ratio of 0.45. They found that mortars made with shell sand exhibited a 17% decrease in compressive strength compared with mortars made with natural sand after 28 days of hardening. They explained that this was owing to the difference in WA between the two types of aggregates. González-Caro et al. [20,26] determined the densities of commercial limestone filler (2654 kg/m^3^) and ATS seashell filler (2613 kg/m^3^) using a helium pycnometer for SC mortar production. Lower densities were obtained in SC mortar made with ATS filler, followed by mixtures made with NA and limestone filler, and finally, NA with SF at a density of 2600 kg/m^3^. Therefore, the density of the filler and porosity also influenced the density of the hardened SC mortar.

Figure 11 shows the APW. The APW rose as the proportion of seashells grew. In the mixtures of Group-1, these increases were 4.67% (Cont-1 vs. 50ATS-1) and 14.00% (Cont-1 vs. 100ATS-1). In the mixtures of Group-2, these increases were 13.49% (Cont-2 vs. 50ATS-2) and 13.64% (Cont-2 vs. 100ATS-2). In the mixtures of Group-3, the increases were 2.76% (Cont-3 vs. 50ATS-3) and 5.60% (Cont-3 vs. 100ATS-3). Cuadrado-Rica et al. [50] observed that incorporating crushed scallop shells as fine aggregates, with replacement levels of up to 60% by mass, led to an increase in the enclused air in fresh concrete. This was attributed to the geometry of the seashell. Eo et al. [51] observed a rise in the air content from 2.2% to 5.6% when 50% of the sand in the concrete was substituted with crushed oyster shells. This increase is linked to the porous characteristics of the oyster shell sand.

Figure 12 presents the WA results, a critical test for assessing the durability of mortar and concrete as it indirectly reflects APW [48]. WA rose in line with the increasing substitution percentage, surpassing the levels observed in the control mixtures. These findings were consistent with the trends seen in APW (Figure 11) and DBD (Figure 10). As bulk density diminished, both porosity and WA exhibited an upward trend.

#### 3.4.4. Dimensional Stability

**Figure 13:**
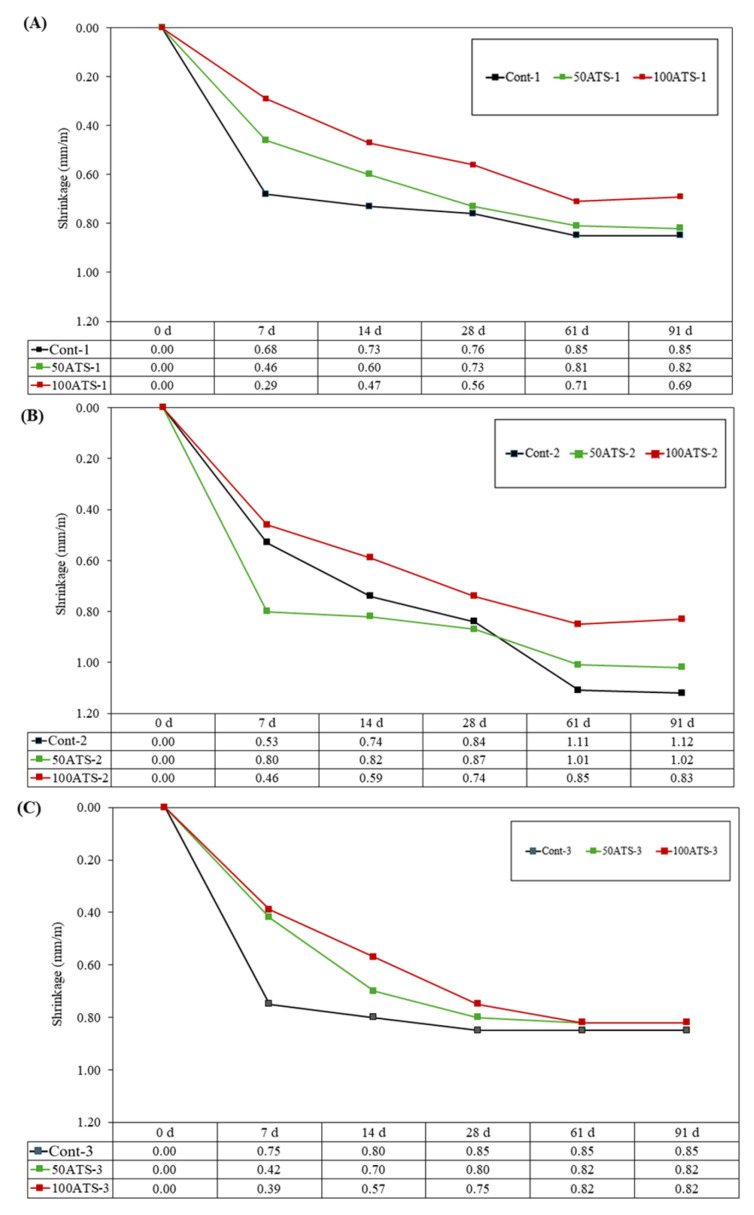
Shrinkages of specimens studied Group-1 (**A**), Group-2 (**B**), and Group-3 (**C**).

Figure 13 illustrates the progression of dimensional stability in mm/m over a period of 91 days for all groups of hardened mortars studied. Dimensional stability decreased across all groups when NA was substituted with 50ATS and 100ATS sands. Significant shrinkage occurred during the first 28 days, but the rate slowed thereafter. The shrinkage test assessed the reduction in volume as the mortar dried and hardened, primarily driven by water loss, which causes contraction in the cement paste. Key factors affecting shrinkage included the w/c ratio and the characteristics of the aggregates.

Mortars containing ATS absorbed more water compared to those with NA, as evident from the higher porosity of ATS sands observed in the SEM images (Figure 3). Consequently, these mixtures had reduced water availability for typical cement hydration reactions. Dimensional stability in mortar is closely tied to water availability, which explains why the higher dimensional stability in the control mixtures was associated with their greater water content. This finding aligned with a dry consistency characteristic observed in the 50ATS and 100ATS mixtures, as shown in Table 2, where both Gm and Rm values declined with increasing ATS content.

Lertwattanaruk et al. [52] examined dimensional stability in mortars where OPC was partially substituted (up to 20%) with mussels, clams, cockles, and oyster shells. Their research revealed that all mortars incorporating seashell powder experienced reduced shrinkage compared to control mortars. This decline was attributed to the seashell particles’ role in pore segmentation, increasing nucleation sites within the spaces between cement particles. This action strengthens the internal structure and lowers overall shrinkage.

#### 3.4.5. MIP

MIP is a commonly employed method for analyzing the pore structure of cementitious materials. In this technique, mercury is injected at high pressure into the material, allowing the pore volume and pore size distribution to be measured.

**Figure 14:**
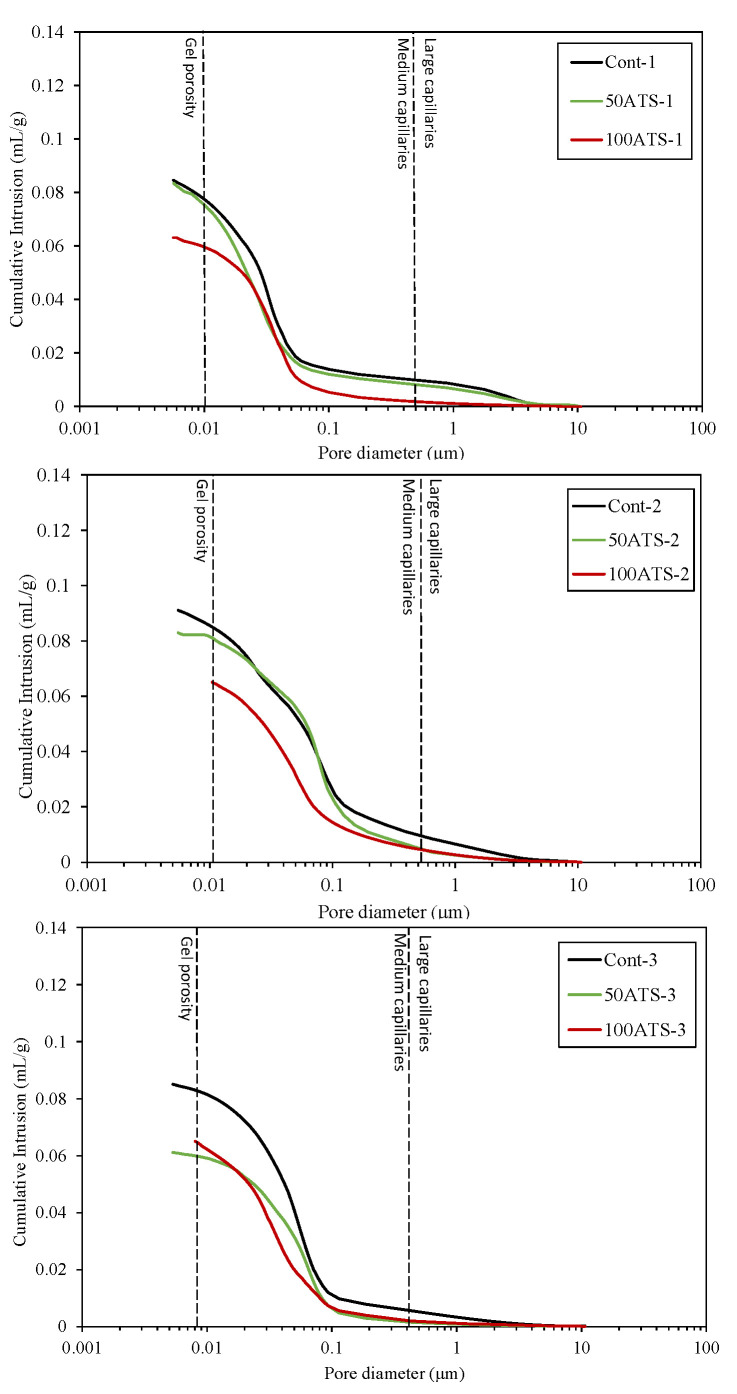
Cumulative pore volumes of specimens studied at 28 days of hardening.

Figure 14 presents the graphical illustration of cumulative mercury intrusion values against pore diameter (μm). Mercury intrusion refers to the volume of mercury invading the pores of a material at different pressure levels. When pressure is applied, mercury begins to invade the pores, and the amount of mercury entering the pores is directly related to their size and accessibility.

The maximum cumulative intrusion volumes recorded for Cont-1, -2, and -3 were 0.084, 0.090, and 0.085 mL/g, correspondingly. Similarly, 50ATS-1, -2, and -3 exhibited peak values of 0.083, 0.082, and 0.061 mL/g, correspondingly. For 100ATS-1, -2, and -3, the highest volumes measured were 0.063, 0.064, and 0.064 mL/g. All the mixtures prepared with 50 and 100ATS had lower total intrusion volumes than the control mixtures. This indicates that the use of ATS seashells occupied the voids and reduced the structure’s porosity. Liao et al. [22] investigated the substitution of river sand with crushed oyster shells in a mortar mixture. They found that mortar containing 30% oyster shell sand had the least porosity. In contrast, the standard mortars displayed higher mercury intrusion, reflecting greater porosity. The cumulative pore volume curves demonstrated a reduction in intrusion values with increasing proportions of oyster shell sand. This suggests that the shell-packing effect reduced the interconnectivity between the pores, leading to a denser mortar structure. Wu et al. [53] conducted a MIP analysis and provided significant insights into the impact of seashell powder (SP) on the pore structures of alkali-activated concrete. Specifically, when 45% SP replaced fly ash, the total porosity decreased by approximately 27%. This decline in porosity results from the micro/nanoparticles of SP filling the voids and acting as initiation sites for hydration products, which improves the internal pore network.

**Figure 15:**
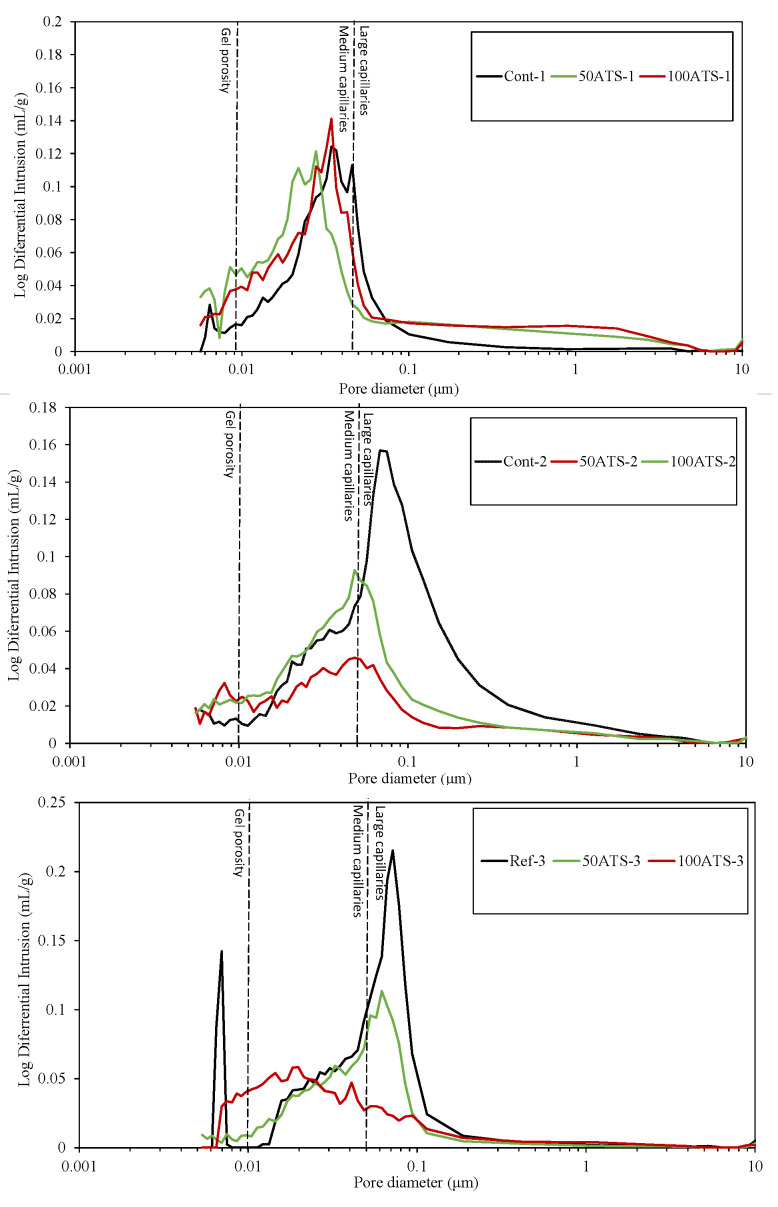
Log differential intrusion (mL/g) of specimens studied.

Figure 15 presents the mercury intrusion values plotted against pore diameter (μm) for the Control, 50ATS, and 100ATS sands across all SC mortars analyzed. These diagrams convey details about the pore size at which mercury starts continuous intrusion (pore frequency). For cement-based materials, they are key for system comparison and percolation assessment [54].

Silva et al. [55] categorized pore sizes as follows: pores with diameters less than 0.01 μm were referred to as “gel porosity”. These pores develop throughout the hydration of the C-S-H gels, correspond to the spaces created between the hydration products, and are not completely accessible for mercury intrusion [21]. Medium capillaries fall within the 0.01–0.05 μm range, while larger capillaries have diameters between 0.05 and 1 μm. Gel porosity impacts properties like shrinkage and consistency, whereas medium capillaries influence mechanical strength, permeability, consistency, and shrinkage. Large capillaries affect both the mechanical strength and permeability [56].

**Figure 16:**
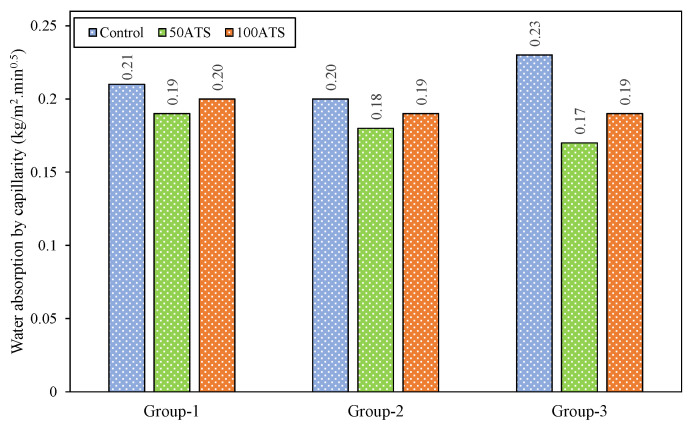
Capillary water absorption specimens were studied at 28 days of hardening.

The results obtained for the pore size were in accordance with the cumulative intrusion volume (Figure 14). For all SC mortar mixtures, the porosity decreased when NA was substituted with the ATS sand. For Cont-1 and 100ATS-1, the utmost pore size was detected at approximately 0.034 μm, and for 50ATS-1 it was 0.027 μm. Cont-2 exhibited the largest pore size of approximately 0.124 μm. However, for 50ATS-2 and 100ATS-2, the pore structure became finer to 0.048 μm. Cont-3 had a pore size of 0.072 μm. For the 50ATS-3 mixture, the pore size decreased by 0.062. For 100ATS-3, there was no clear peak of maximum pore size, and the maximum pore distribution was within the medium capillaries. All the SC mortar groups made with ATS sands were found to be within the medium capillary range, except for 50ATS-3. The use of seashells decreased porosity. These results were in accordance with other durability properties, such as shrinkage (Figure 13) and capillary water absorption (Figure 16), which were enhanced by the use of the ATS sand.

The reduction in porosity due to the use of shells has also been explained by other authors. The authors attributed this to the packing effect of the shells in the mortar, which reduced the number of pores. In a previous study, Liao et al. [22] reported a reduction in both pore diameter and porosity when increasing amounts of oyster shell sand were used as substitutes (up to 30%). This adjustment led to the development of a finer pore structure in the modified mortar than in the control, resulting in a more compact material. Similarly, Safi et al. [44] examined the inclusion of 50% and 100% waste seashell sand in SC mortar. Their findings revealed that, as the proportion of seashell sand increased, the elongated geometry of the particles facilitated the filling of voids, which in turn decreased the porosity of the mortar.

#### 3.4.6. Water Absorption by Capillarity

Cement-based materials are known for their porous structures, which generally appear in two forms. One form consists of isolated pores located at the boundary layer between the cement paste and aggregates, with the other featuring interconnected pores that are open to the surface, forming a network of channels within the internal structure of the matrix. Capillary water absorption is associated with the connectivity of the internal pores, which can lead to the capillary movement of water or other substances. This effect may be detrimental in certain contexts where it is directly influenced by the coefficient. The interconnected pores generate a water flow that affects the durability of the mortars [57].

Figure 16 presents graphical representations of water absorption by capillarity for all the groups of specimens studied at 28 days of hardening. Capillary water absorption decreased when the natural sand was replaced by ATS sands in all groups. The percentage decreases compared with the control mixtures were 9.52%, 10.00%, and 26.09% for 50ATS-1, 50ATS-2, and 50ATS-3, correspondingly, and 4.76%, 5.00%, and 17.39% for 100ATS-1, 100ATS-2, and 100ATS-3, correspondingly. SC mortars manufactured with 50ATS and 100ATS sand seashells showed lower absorption than those made with natural sand. This means that the SC mortar made with the ATS sand has fewer interconnections between the pores. The uneven and porous surface of the ATS seashell blocked the interconnections between the pores. This is a significant advantage in terms of durability.

Martínez-García et al. [58] investigated water absorption by capillarity in mortars incorporating mussel shell sand and observed a reduction in water absorption as the proportion of shell substitution increased. This was attributed to the elongated geometry of the mussel shell particles, which served as a protection against water intrusion in the mortar. In another study, these authors [59] investigated the limit of mussel sand substitution in mortars with up to 75% substitution. They concluded that the use of shells created tortuous pathways for water, preventing the migration of water through capillary pores.

## 4. Conclusions

The potential use of ground ATS seashells as recycled sand in an SC mortar was assessed experimentally. Derived from the findings, the following conclusions were established:

The use of ATS recycled sand in self-compacting mortars (SC mortar) resulted in a drier consistency due to the porous, angular, and uneven surfaces of the particles, as confirmed by SEM analysis, which also highlights their higher porosity compared to quarried aggregates.

XRD analysis revealed that, despite no new phases forming in mortars with ATS, the increased porosity facilitates greater calcite formation after 91 days of curing by providing additional nucleation sites during cement hydration.

Mechanical properties were slightly reduced at 50% and 100% replacement levels, attributed to the higher water absorption of ATS sand and a lower effective water-to-cement ratio. However, compressive strength values exceeded 40 MPa, which is sufficiently high for manufacturing construction elements using self-compacting material technology.

Durability tests indicated reduced shrinkage with ATS sand, as the unique geometry of the particles collapses small capillary pores (0.1–1 μm) and limits interconnected porosity, though the formation of larger pores (>1 μm) and increases water absorption, apparent porosity, and diminishes mechanical strength and bulk densities.

Overall, the use of recycled sand from ATS seashells enhances the durability of SC mortar compared to mortars with natural sand and provides an environmentally friendly solution by reducing the disposal of seashell waste in landfills and decreasing the extraction of natural aggregates from quarries.

Future research on ATS recycled sand should focus on optimizing grinding processes to produce more uniform and less porous particles, exploring additives to reduce water absorption, while enhancing nucleation benefits, and conducting long-term durability studies to assess properties like freeze-thaw resistance and chemical attack. The analysis by scanning electron microscopy and their correlations with macroscopic mechanical properties will enhance the knowledge of the behavior of these SC mortars. Additionally, assessing the carbon footprint and environmental impact of using recycled shells in mortars, and highlighting their benefits over traditional materials. These strategies aim to improve mechanical performance, durability, and sustainability, supporting the effective integration of ATS sand into environmentally friendly construction practices.

## Figures and Tables

**Table 1:** Terminology, mixture quantities (kg/m^3^), and workability parameters of SC mortar.

		Group-1			Group-2			Group-3		
		Cont-1	50ATS	100ATS	Cont-2	50ATS	100ATS	Cont-3	50ATS	100ATS
Powders	CEM	490.0	490.0	490.0	525.4	525.4	525.4	556.1	556.1	556.1
	Siliceous Filler (SF)	293.9	293.9	293.9	315.1	315.1	315.1	333.5	333.5	333.5
Fine aggregates	NA-0/3	617.0	308.5	0	567.0	283.5	0	525.2	262.6	0
	NA-0/6	617.9	310.8	0	567.9	285.7	0	526.0	264.6	0
	ATS sand	0	641.2	1282.5	0	589.3	1178.6	0	545.8	1091.6
	Water	240.2	240.2	240.2	257.5	257.5	257.5	272.6	272.6	272.6
Workability parameters	Vp/Vs	0.6	0.6	0.6	0.7	0.7	0.7	0.8	0.8	0.8
	Vw/Vp	0.85	0.85	0.85	0.85	0.85	0.85	0.85	0.85	0.85
	Sp/p %	0.84	0.84	0.84	0.84	0.84	0.84	0.69	0.69	0.69

Vp/Vs (volume of powder materials/volume of fine aggregates); Vw/Vp (volume of water/volume of powder materials); Sp/p % (percentage of superplasticiser relative to powder materials).

**Table 2:** Workability parameters of the specimens studied.

	Group-1			Group-2			Group-3		
	Cont-1	50ATS-1	100ATS-1	Cont-2	50ATS-2	100ATS-2	Cont-3	50ATS-3	100ATS-3
Gm	5.25	5.00	4.86	5.50	5.25	4.90	5.76	5.55	5.25
Rm (s^−1^)	1.25	1.33	1.43	1.11	1.18	1.25	1.00	1.05	1.11

## Data Availability

The original contributions presented in the study are included in the article, further inquiries can be directed to the corresponding authors.

## References

[1] Chen B., Peng L., Zhong H., Zhao Y., Meng T., Zhang B. (2023). Improving the Mechanical Properties of Mussel Shell Aggregate Concrete by Aggregate Modification and Mixture Design. Case Stud. Constr. Mater..

[2] Bampanis I., Vasilatos C. (2023). Recycling Concrete to Aggregates. Implications on CO_2_ Footprint. Mater. Proc..

[3] Market C.I., Persistence Market Research Healthcare Cloud Computing Market—Global Study on Healthcare Cloud. https://www.persistencemarketresearch.com/.

[4] FAO (2024). The State of World Fisheries and Aquaculture 2024—Blue Transformation in Action.

[5] COM(2020)98, 2020. European Commission, Report from the Commission to the European Parliament, the Council, the European Economic and Social Committee and the Committee of the Regions on the Implementation of the Circular Economy Action Plan. https://environment.ec.europa.eu/strategy/circular-economy-action-plan_en.

[6] Bellei P., Torres I., Solstad R., Flores-Colen I. (2023). Potential Use of Oyster Shell Waste in the Composition of Construction Composites: A Review. Buildings.

[7] Eziefula U.G., Ezeh J.C., Eziefula B.I. (2018). Properties of Seashell Aggregate Concrete: A Review. Constr. Build. Mater..

[8] Asaoka S., Yamamoto T., Kondo S., Hayakawa S. (2009). Removal of Hydrogen Sulfide Using Crushed Oyster Shell from Pore Water to Remediate Organically Enriched Coastal Marine Sediments. Bioresour. Technol..

[9] Olivia M., Oktaviani R. (2017). Properties of Concrete Containing Ground Waste Cockle and Clam Seashells. Procedia Eng..

[10] Martínez-García C., González-Fonteboa B., Martínez-Abella F., López D.C. (2017). Performance of Mussel Shell as Aggregate in Plain Concrete. Constr. Build. Mater..

[11] Park W.H., Polprasert C. (2008). Roles of Oyster Shells in an Integrated Constructed Wetland System Designed for P Removal. Ecol. Eng..

[12] Richardson A.E., Fuller T. (2013). Sea Shells Used as Partial Aggregate Replacement in Concrete. Struct. Surv..

[13] Li G., Xu X., Chen E., Fan J., Xiong G. (2015). Properties of Cement-Based Bricks with Oyster-Shells Ash. J. Clean. Prod..

[14] Kuo W.-T., Wang H.Y., Shu C.Y., Su D.S. (2013). Engineering Properties of Controlled Low-Strength Materials Containing Waste Oyster Shells. Constr. Build. Mater..

[15] Agbede O.I., Manasseh J. (2009). Suitability of Periwinkle Shell as Partial Replacement for River Gravel in Concrete. Leonardo Electron. J. Pract. Technol..

[16] Nakatani N., Takamori H., Takeda K., Sakugawa H. (2009). Transesterification of Soybean Oil Using Combusted Oyster Shell Waste as a Catalyst. Bioresour. Technol..

[17] Olivia M., Mifshella A.A., Darmayanti L. (2015). Mechanical Properties of Seashell Concrete. Procedia Eng..

[18] Zhang Y., Chen D., Liang Y., Qu K., Lu K., Chen S., Kong M. (2020). Study on Engineering Properties of Foam Concrete Containing Waste Seashell. Constr. Build. Mater..

[19] Tayeh B.A., Hasaniyah M.W., Zeyad A.M., Yusuf M.O. (2019). Properties of Concrete Containing Recycled Seashells as Cement Partial Replacement: A Review. J. Clean. Prod..

[20] González-Caro Á., Merino-Lechuga A.M., Fernández-Ledesma E., Fernández-Rodríguez J.M., Jiménez J.R., Suescum-Morales D. (2023). The Effect of Acanthocardia Tuberculata Shell Powder as Filler on the Performance of Self-Compacting Mortar. Materials.

[21] Liao Y., Fan J., Li R., Da B., Chen D., Zhang Y. (2022). Influence of the Usage of Waste Oyster Shell Powder on Mechanical Properties and Durability of Mortar. Adv. Powder Technol..

[22] Liao Y., Wang X., Kong D., Da B., Chen D. (2023). Experiment Research on Effect of Oyster Shell Particle Size on Mortar Transmission Properties. Constr. Build. Mater..

[23] Yang E.I., Yi S.T., Leem Y.M. (2005). Effect of Oyster Shell Substituted for Fine Aggregate on Concrete Characteristics: Part I. Fundamental Properties. Cem. Concr. Res..

[24] Billberg P. (1999). Fine Mortars Rheology in Mix Design of SCC. First International RILEM Symposium on Self-Compacting Concrete.

[25] Nepomuceno M., Oliveira L., Lopes S.M.R. (2012). Methodology for Mix Design of the Mortar Phase of Self-Compacting Concrete Using Different Mineral Additions in Binary Blends of Powders. Constr. Build. Mater..

[26] González-Caro Á., Merino-Lechuga A.M., Fernández-Ledesma E., María Fernández-Rodríguez J., Ramón Jiménez J., Suescum-Morales D. (2024). Use of Milled Acanthocardia Tuberculate Seashell as Fine Aggregate in Self-Compacting Mortars. Materials.

[27] EFNARC (2002). EFNARC Specification and Guidelines for Self-Compacting Concrete.

[28] (2014). Concrete Durability. Test Methods. Determination of the Water Absorption, Density and Accesible Porosity for Water in Concrete.

[29] Centro de Investigación, Tecnología e Innovación de La Universidad de Sevilla (CITIUS). https://citius.us.es/web/.

[30] (2021). Mortars. Methods of Test for Hardened Mortar for Mansory—Determination of Dimensional Stability of Hardened Mortar for Mansory.

[31] (2003). Methods of Test for Mortar for Mansory—Part 18: Determination of Water Absorption Coefficient Due to Capillary Action of Hardened Mortar.

[32] Merino-Lechuga A.M., González-Caro Á., Fernández-Ledesma E., Jiménez J.R., Fernández-Rodríguez J.M., Suescum-Morales D. (2023). Accelerated Carbonation of Vibro-Compacted Porous Concrete for Eco-Friendly Precast Elements. Materials.

[33] Mohit M., Haftbaradaran H., Riahi H.T. (2023). Investigating the Ternary Cement Containing Portland Cement, Ceramic Waste Powder, and Limestone. Constr. Build. Mater..

[34] Mo K.H., Alengaram U.J., Jumaat M.Z., Lee S.C., Goh W.I., Yuen C.W. (2018). Recycling of Seashell Waste in Concrete: A Review. Constr. Build. Mater..

[35] JCPDS (1970). Joint Committee on Powder Diffraction Standards. Anal. Chem..

[36] Suescum-Morales D., Fernández-Ledesma E., González-Caro Á., Merino-Lechuga A.M., Fernández-Rodríguez J.M., Jiménez J.R. (2023). Carbon Emission Evaluation of CO_2_ Curing in Vibro-Compacted Precast Concrete Made with Recycled Aggregates. Materials.

[37] Suescum-Morales D., Silva R.V., Bravo M., Jiménez J.R., Fernández-Rodríguez J.M., de Brito J. (2022). Effect of Incorporating Municipal Solid Waste Incinerated Bottom Ash in Alkali-Activated Fly Ash Concrete Subjected to Accelerated CO_2_ Curing. J. Clean. Prod..

[38] Kalinowska-Wichrowska K., Pawluczuk E., Bołtryk M., Jimenez J.R., Fernandez-Rodriguez J.M., Morales D.S. (2022). The Performance of Concrete Made with Secondary Products—Recycled Coarse Aggregates, Recycled Cement Mortar, and Fly Ash–Slag Mix. Materials.

[39] Murthy I.N., Rao J.B. (2016). Investigations on Physical and Chemical Properties of High Silica Sand, Fe-Cr Slag and Blast Furnace Slag for Foundry Applications. Procedia Environ. Sci..

[40] Perić J., Vueak M., Krstulovib R., Breeevib L., Kralj D. (1996). Phase Transformation of Calcium Carbonate Polymorphs. Thermochim. Acta.

[41] Barros M.C., Bello P.M., Bao M., Torrado J.J. (2009). From Waste to Commodity: Transforming Shells into High Purity Calcium Carbonate. J. Clean. Prod..

[42] Mohamed M., Rashidi N.A., Yusup S., Teong L.K., Rashid U., Ali R.M. (2012). Effects of Experimental Variables on Conversion of Cockle Shell to Calcium Oxide Using Thermal Gravimetric Analysis. J. Clean. Prod..

[43] Hadjadj M., Guendouz M., Boukhelkhal D. (2024). The Effect of Using Seashells as Cementitious Bio-Material and Granite Industrial Waste as Fine Aggregate on Mechanical and Durability Properties of Green Flowable Sand Concrete. J. Build. Eng..

[44] Safi B., Saidi M., Daoui A., Bellal A., Mechekak A., Toumi K. (2015). The Use of Seashells as a Fine Aggregate (by Sand Substitution) in Self-Compacting Mortar (SC mortar). Constr. Build. Mater..

[45] Oh S.E., Chung S.Y., Kim K., Han S.H. (2024). Comparative Analysis of the Effects of Waste Shell Aggregates on the Material Properties of Cement Mortars. Constr. Build. Mater..

[46] Lozano-Lunar A., Dubchenko I., Bashynskyi S., Rodero A., Fernández J.M., Jiménez J.R. (2020). Performance of Self-Compacting Mortars with Granite Sludge as Aggregate. Constr. Build. Mater..

[47] Nduka D.O., Akanbi E.T., Ojo D.O., Babayemi T.E., Jolayemi K.J. (2023). Investigation of the Mechanical and Microstructural Properties of Masonry Mortar Made with Seashell Particles. Materials.

[48] Suarez-Riera D., Merlo A., Lavagna L., Nisticò R., Pavese M. (2021). Mechanical Properties of Mortar Containing Recycled Acanthocardia Tuberculata Seashells as Aggregate Partial Replacement. Bol. Soc. Esp. Ceram. Vidr..

[49] Edalat-Behbahani A., Soltanzadeh F., Emam-Jomeh M., Soltan-Zadeh Z. (2021). Sustainable Approaches for Developing Concrete and Mortar Using Waste Seashell. Eur. J. Environ. Civ. Eng..

[50] Cuadrado-Rica H., Sebaibi N., Boutouil M., Boudart B. (2016). Properties of Ordinary Concretes Incorporating Crushed Queen Scallop Shells. Mater. Struct./Mater. Constr..

[51] Eo S.H., Yi S.T. (2015). Effect of Oyster Shell as an Aggregate Replacement on the Characteristics of Concrete. Mag. Concr. Res..

[52] Lertwattanaruk P., Makul N., Siripattarapravat C. (2012). Utilization of Ground Waste Seashells in Cement Mortars for Masonry and Plastering. J. Environ. Manag..

[53] Wu Y., Lu J., Nie Y., He W. (2024). Effect of Seashell Powder as Binder Material on the Performance and Microstructure of Low-Carbon Sustainable Alkali-Activated Concrete. J. Build. Eng..

[54] Abell A.B., Willis K.L., Lange D.A. (1999). Mercury Intrusion Porosimetry and Image Analysis of Cement-Based Materials. J. Colloid Interface Sci..

[55] Silva D.A., John V.M., Ribeiro J.L.D., Roman H.R. (2001). Pore Size Distribution of Hydrated Cement Pastes Modified with Polymers. Cem. Concr. Res..

[56] López-Uceda A., Cantador-Fernández D., Da Silva P.R., de Brito J., Fernández-Rodríguez J.M., Jiménez J.R. (2024). Mechanical and Durability Performance of Self-Compacting Mortars Made with Different Industrial by-Products as Fillers. Constr. Build. Mater..

[57] Baptista Junior G., Nascimento L.C., de Xavier G.C., Monteiro S.N., Vieira C.M.F., Marvila M.T., Alledi C.T.D.B. (2024). Durability for Coating Mortars: Review of Methodologies. J. Mater. Res. Technol..

[58] Martínez-García C., González-Fonteboa B., Carro-López D., Martínez-Abella F. (2020). Effects of Mussel Shell Aggregates on Hygric Behaviour of Air Lime Mortar at Different Ages. Constr. Build. Mater..

[59] Martínez-García C., González-Fonteboa B., Carro-López D., Martínez-Abella F. (2024). Mussel Shell Mortars Durability: Study of Aggregate Replacement Limit. J. Build. Eng..

